# On improving public health after COVID-19 epidemic: A fractal-fractional mathematical solutions with short memory effect and efficient optimal strategies

**DOI:** 10.1371/journal.pone.0321195

**Published:** 2025-05-28

**Authors:** Biplab Dhar, Mohammad Sajid

**Affiliations:** 1 Applied Science Cluster—Mathematics, UPES Dehradun, Uttarakhand, India; 2 Department of Mechanical Engineering, College of Engineering, Qassim University, Buraydah, Saudi Arabia; China University of Mining and Technology, CHINA

## Abstract

As per the report of W.H.O. about 7 million people died in India till date due to COVID-19 infection. The transmission of COVID-19 infection can affect the temporal and geographic diversity of environmental pollution, thereby disrupting “planetary health” and livelihood. The consensus is that COVID-19 could have significant long-lasting effects on ecosystem and society. It is possible to reach an agreement to create and maintain an ecologically sound environment and a circular bio-economy to try to solve these issues. For the first time, a fractional mathematical model is formulated where the infection is considered due to unhygienic environment with a synergy between mathematical fractal parameters and biology of the disease transmission. Other mathematical analysis such as the boundedness of solutions, the wellposedness of the proposed model concerning existence results, etc. are investigated. Additionally, evaluation of vaccine-clearance equilibrium point is performed. Sensitivity parameters analysis and model’s stability also steps in. To get numerical results, the “Adams–Bashforth–Moulton” method with slight modification in the kernel is used. The fractional parameters: memory effect and fractional diffusion shows a good performance of the proposed model in depicting the disease dynamics. Consequences of follow-up optimal control functions in Susceptives and Vaccinated individuals, where feasible strategies in terms of the control maps are presented.

## Introduction

One perspective on the Corona-virus infections of 2019 (COVID-19) could center on the vector-borne spread linked to the Plantationocene’s declining biodiversity and deteriorating biotopes [1, 2]. COVID-19 transmission is closely associated with environmental sustainability. By undermining international efforts to mitigate immediate threats to natural resources, the spread of this disease has also impeded progress toward achieving the “Sustainable Development Goals" of the United Nations. It has also had a negative impact on people’s health, livelihood, forestry, agriculture, and international peace [3]. Following the first infection case, lock-downs around the world had both beneficial and negative effects on the environment and climate. Interestingly, because of the lock-downs there were only temporarily restrictions on plantationogenic activities and the beneficial effects on the ecosystem are not long-lasting [4]. Recent years have seen a significant reliance on empirical research for the manifestations and management of COVID-19. For instance, although early trends suggested a decrease in environmental pollution, there is not enough information to evaluate the long-term ecological consequences. The long-term view has also been neglected in evaluations of the early impacts of COVID-19 and its developing variations on the spatial environment recovery during global lock-downs [5]. The development of policies targeted at mitigating the epidemic has not yet fully sustainably integrated, despite global efforts to address the issue. Numerous studies indicate that nations with sub-par or mediocre healthcare systems experienced lock-downs more frequently and compulsively. Despite precautionary measures like lockdowns and vaccinations, a few factors were critical in the epidemiology of COVID-19 infections such as environment, season, climate, etc. [6]. Temperature and humidity are two meteorological parameters that are closely related to its spread and the hazards to human health. Consequently, rather than being referred to as “human-to-human" transmission, the consequences of COVID-19’s transmission dynamics are identified as “air pollution-to-human". As a result, it is recognized that gases and particulate matter (PM) play a major role as active media in the spread of infections and the death that goes along with them. A clear or comprehensive summary of the worldwide problem of combining workable, sustainable, and ecosystem-based solutions can be obtained through the visualization of several sectors. By tracing the connections between “nexus" indicators and the impacts of COVID-19, this research article is anticipated to contribute to the enhancement of planetary health [7].

### Effects of COVID-19 on nature

Unprecedented attention has been paid to the consequences and aftermath of COVID-19, especially in relation to environment quality. Lock-downs aimed at curbing the transmission of the virus are accountable for the deterioration of the environment by producing a greater quantity of bio-medical waste (<400%), non-biodegradable waste (≈200%), ozone depletion (<60%), and pollutants [8]. In general, the COVID-19 epidemic’s deteriorating environmental quality was only temporarily improved by lower industrial emissions; however, the negative consequences of human efforts to contain the virus are probably going to last longer.

#### Quality of air.

Changes in air quality indices, such as ambient PM10/PM2.5 levels, air pollutants, and local weather patterns, were closely associated with the spread of COVID-19. Similar reductions in PM levels of about 71% were noted in a number of India’s largest cities [6]. As the spread of COVID-19 aerosols can be influenced by meteorological factors, the associated health risks are closely linked to air quality. Worldwide, there has been a notable increase in the infection and mortality rates in cities with poor air quality [9]. Duration of the virus’s residence in atmospheric components is another important factor that impacts the COVID-19 epidemiology. Public health officials and epidemiologists faced difficulties as a result of the heightened its disease and death rates caused by air pollution in cities and related climate factors. In this regard, it was found that COVID-19 infections were higher in hinterlands than in coastal areas.

#### Aquatic ecosystem and science of safety.

Coral reefs and other marine ecosystems were said to have at least partially recovered during lock-downs [10]. Significantly, the water condition of ecosystems that use freshwater, like the Ganga River, was also improved. As a result, COVID-19 made it possible to revitalize aquatic ecosystems using “nature-based solutions" [11]. Conversely, it appears that the virus’s extended persistence in poorly maintained sewer treatment facilities was released in aerosol form from wastewater pathways causing disturbances in aquatic ecosystems. Municipal waste treatment facilities and wastewater can serve as the main hubs for COVID-19 infection. The virus level in wastewater can be reduced by widely applying conventional disinfectants.

#### Climate.

This epidemic had an impact on climate change, which are closely related to the disease’s epidemiology. Human health risks are directly linked to the synergy created by COVID-19 and co-existing climate variables. However, because COVID-19-specific medical facilities and medical sciences are given priority, climate change action research is frequently disregarded. In actuality, the health risks associated with an increase in the global average temperature of more than 1.5^∘^C could be even more disastrous than those brought on by COVID-19 [12]. Further efforts to address the impact of climate change on the epidemic are needed to help lower the risks associated with COVID-19. Since then, the disease has served as a reminder to international organizations to coordinate their efforts in order to address the detrimental effects of climate change. It may be too soon to tell how lock-downs affected various environmental matrices, the transportation and industrial sectors, and the global climate.

#### Planetary well-being and sustenance.

Global public health was severely disrupted by the COVID-19 epidemic in terms of both mortality and morbidity. The W.H.O estimated that as of 11 June 2024 over 7.1 million people had died from COVID-19, whilst approximately 776 million people had been infected and experienced severe cardiac and respiratory issues, and 5470 million vaccinated [13]. Lung tissues are the primary target of the virus’s variants in humans, with a wide range of effects documented. The severity of COVID-19 is influenced not only by general human health indicators but also by socioeconomic, environmental, clinical, and unique genetic factors of infected hosts. Thus, following the infection, “autoimmune and inflammatory" disease actions are thought to be correlated with the genetic composition of the human population [14]. It has been established that the infection poses health risks due to molecular disturbance of lung thrombosis. Certain COVID-19 variations have a number of negative health effects that can combine to cause lung apoptosis which may lead to serious respiratory issues.

The potential hazards of PM2.5 from domestic cooking in countries with middle and low incomes can further raise the health risks for COVID-19 patients. The disease can exacerbate neurological and mental illnesses in addition to its direct effects on the heart and lungs [15]. In addition, it can cause anxiety and stress signs in infected patients and worsen pre-existing psychological neural illnesses in non-infected people. Under COVID-19, psychological or mental distress can lower immunity and cause cardiac abnormalities, so it’s important to give related medical research top priority [16].

#### The “nexus" viewpoint.

According to the nexus perspective, human disruption of “land use–food–wildlife” nexus of the Rhinolophidaes may have resulted in the virus outbreak. However, if other issues are not resolved, efforts to lessen the specific environmental effects of COVID-19 might not be sustainable. Research findings indicate that implementing linear and monocentric methods does not improve long-term environmental resilience or sustainability, nor does it lessen the impact of COVID-19. A nexus viewpoint can be very helpful for ecosystem-based viable approaches to mitigate the effects of the epidemic [18].

### Mathematics as symbiote

**Fig 1:**
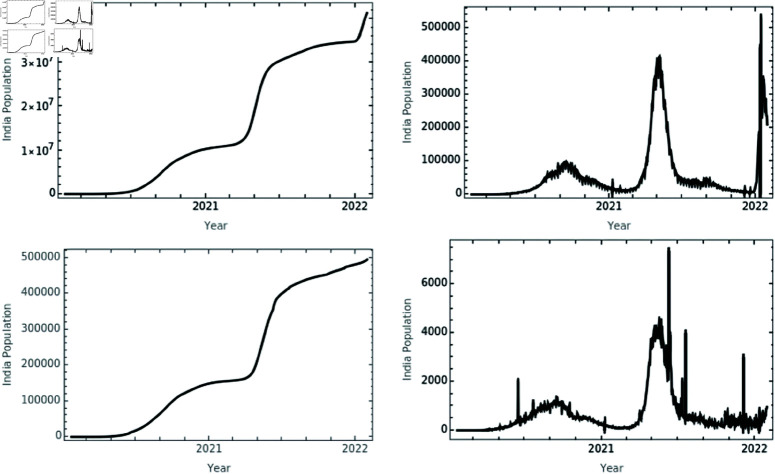
India COVID-19 statistical survey. The figure depicts on the number of confirm cases, daily confirm cases (first row; left to right): death cases, and daily death cases (second row; left to right) taken from “Wolfram Function Repository" [17].

With the advent of the COVID-19 vaccine and non-pharmaceutical measures like social distancing, self-isolation, face masks, hand globes, and overall gowns, people’s awareness needs to be raised to stop the disease from spreading. The Indian government acted quickly to take preventive and control measures to stop the virus spread. The Fig 1 portray the number confirmed cases of COVID-19 and deaths caused in India till the year 2022. The primary causes of the local COVID-19 prevalence in mainland India were the importation of infection cases, population mobility, and variant evolution. The Indian government imposed lock-down, normalization stage prevention and control, and lock-down with least amount of possible loss against COVID-19. In the global context, mathematical model assessment proved to be one of the most useful methods for analyzing the spread of epidemics. In particular, SEIR frameworks were extensively used to investigate scenario studies and the long-term actions of the transmission mechanism [19–22]. In these studies, the effects of vaccination, non-pharmacological interventions, and variant evolution were thoroughly examined but missed out proper planning or strategies to contain the infection spread.

Infectious disease research heavily relies on mathematical modeling. The trends and variations in infectious diseases such as influenza virus, AIDS, TB, HIV, etc. have been studied with this method [23–26]. The interconnection of state variables of a diseases can be depicted through mathematical models by including parameters such as traits of the disease, population rates, and association rates. Based on data availability, these models carefully foresee and anticipate the onset of diseases and assess the effectiveness of various strategies, including medication, isolation, vaccination, and quarantine. There are many uses for mathematical frameworks, including disease staging rates, medical access, and demographic shifts. These tactics aid in the prevention or curing of illness. In recent years, a number of helpful mathematical simulations have been created to analyze the epidemic and create practical plans for the rapid eradication of infection [27–32]. Mathematical modeling serves educational, informative, and actual purposes by establishing connections between mathematical ideas and real-world phenomena.

Fractional calculus is gaining attention from researchers all over the world due to its numerous advantages and practical applications to physics and engineering issues. This is because fractional calculus has potential applications beyond mathematics [33, 34]. A model with a fractional-order system may be the only one suitable for studying memory, switching behavior, and genetic variation [35]. Atangana [36] developed “fractal-fractional differential and integral operators" that are used to quantify the fractal dimensions of real events. When dealing with complex physical problems, like those that display fractal characteristics, the “fractal-fractional" operator (call it as F-F operator) is utilized to perform a single differentiation by combining fractional differentiation with fractal derivative. The F-F mathematical models offer a fascinating and sophisticated framework for studying complex and self-repeating fluctuations in a range of systems. These models embrace the concept of fractional dimensions in lieu of normal mathematical models, which typically rely on integer dimensions and continuous functions. Utilizing Atangana–Baleanu and Caputo derivatives, a mathematical model of listeriosis integrates F-F ordering, enabling researchers to study the disease’s memory-related future behaviors. Numbered results are provided for each F-F order operator, along with the steady states and listeriosis transmission threshold parameters [37]. An Atangana–Baleanu operator was utilized for an F-F computer malware system. The paper employs fixed-point theorems to confirm uniqueness, utilizes Atangana-Toufik approaches to obtain approximate solutions, and employs visual aids to examine the efficacy of the method in varying orders and initial conditions [38]. A theory of fixed point was used to construct Finite-Time Stability in a fractional order system, in contrast to the methods that depended on the “Gronwall inequality". There are also two examples provided to support and validate the theoretical contributions [39]. The investigation of therapy, immunization, a second infection, and asymptomatic recipients in dengue illness transmission was done using fractional calculus. The model’s Ulam–Hyers stability was investigated by examining the dengue infection’s dynamic behavior and qualitative methodology. The L-A decomposition approach was utilized to analyze the solution pathways [40]. Another study looked into the multidisciplinary stages of nonlinear research, emphasizing the challenges of coming up with intricate, creative processes and assuming organized mathematical frameworks. It investigates the application of fractional differential equations and neural networks to chaotic and nonlinear systems. This special issue brings together cutting-edge research from a range of fields to present improved understanding and models for collected fractional mathematical problems [41]. One study that looked at the relationship between HIV viruses and HIV-positive or HIV-negative T-cells developed an original framework for the HIV transmission dynamics in vivo. The system’s solution paths and chaotic behavior were numerically illustrated, and it was explored in terms of Liouville-Caputo and Atangana–Baleanu fractional operators. It was found that system outputs could affect the fractional parameter [42]. Atangana–Baleanu fractional derivative was used to develop a mathematical model to study the spread of tungiasis infection. It considered how people and sand fleas interacted, including infection rate, incubation period, and recovery rate. Through numerical simulations, the impact of management approaches and treatments on tungiasis prevalence was evaluated [43]. By incorporating memory weight and non-Markovian behavior, F-F disease simulations are able to more accurately represent the enduring and continuing dynamics observed in infectious diseases [35, 44–46].

Optimal control is an efficient mathematical tool used in many fields, including robotics, economics and finance, aeronautic engineering, epidemiology, and many more, to optimize control problems in the respective fields [47, 48]. The majority of numerical representations of COVID-19 that have been studied in the literature did not take into account the most practical approaches, which are time-dependent control strategies. Therefore, programs for controlling epidemics can be designed or suggested using this approach [49, 50]. Numerous studies for optimal control take into account various factors depending on the geography and economy of the location such as India [51], Nigeria [52], and Portugal [53]. Mathematical schemes working under fractional order derivatives may fully characterize the local and global dynamics of COVID-19. These kinds of models are also better at precisely and accurately representing observed phenomena because they utilize fractional calculus [54–57]. In order to create the best manages for vaccination or constraints strategies, many researchers created COVID-19 mathematical frameworks. Although, irrespective of vaccination protocols and control measures it can be concluded that seasonal and environmental factors do influence COVID-19 transmission [58].

The remaining parts of the article are catalogued as follows: Definitions are provided in the Sect Methodology to understand the F-F operator. The model is then formulated under certain assumptions, which is presented in Sect The model: Formulation and assumptions including a flow-diagram. The biological significance of the proposed model and two fractional parameters are also discussed elaborately in this section. This section also has full model analysis stating existence-uniqueness, non-negativity, and boundedness of solutions for state variables. The evaluation of vaccine-clearance equilibrium point is also present in this section. The Sect The basic and the effective reproduction number is comprised of detailed aspects of the basic reproduction number and the effective reproduction number with graphical representations. Following which, the sensitivity analysis is included for both the basic and the effective reproduction number in Sect Sensitivity analysis. The Ulam–Hyers (U-H) stability of the model is done in Sect U-H stability. A detailed analysis for optimal control strategies is done in Sect Optimal control strategies together with the formation and existence of the controls. The control functions are then further classified in terms of strategies. The efficiency analysis for strategies is also done in this section. The numerical simulations and results for different cases related to non-pharmacological interventions, controls, and strategies are noted in Sect Numerical simulation and results together with graphical representations. In Sect Discussions, discussions are written on basis of the findings keeping scientific notion alive, followed by conclusions in Sect Conclusions.

## Methodology

A few basic definitions relevant to F-F operators are covered in this section, which are adapted from the references [36, 59]. It is assumed that the set {𝒳(t)∈𝒞([0,1])→ℝ} has a norm defined as $∥𝒳(t)∥=\max_{t\in \left[0,1\right]}|𝒳(t)|$.

**Definition 1.** *Let $𝒳(t)\in (x_{1},x_{2})$ be a fractional differentiable smooth curve is present in the sense 0<τ<1 and 0<ρ≤1, then F-F Atangana–Baleanu (AB) derivative sense with generalized “Mittag-Lefller” (M-L) kernel*


$${\;}^{ff}{𝒟}_{x_{1},t}^{\tau ,\rho }𝒳(t)=\frac{𝒩(\tau )}{1-\tau }\frac{d}{dt^{\rho }}\int_{x_{1}}^{t}𝒳(s){ℰ}_{\tau }\left[-\frac{\tau }{1-\tau }(t-s{)}^{\tau }\right]ds,$$

*where $𝒩(\tau )=1-\tau +\frac{\tau }{\Gamma (\tau )}$ is called normalization map satisfying 𝒩(0)=𝒩(1)=1, can be generalized into*


$${\;}^{ff}{𝒟}_{x_{1},t}^{\tau ,\rho ,a}ℱ(t)=\frac{𝒩(\tau )}{1-\tau }\frac{d^{a}}{dt^{\rho }}\int_{x_{1}}^{t}ℱ(s){ℰ}_{\tau }\left[-\frac{\tau }{1-\tau }(t-s{)}^{\tau }\right]ds,$$

*where a∈(0,1]. Here, $\frac{d𝒳(s)}{ds^{\rho }}=\lim_{b\to s}\frac{𝒳(b)-𝒳(s)}{b^{\rho }-s^{\rho }}$.*

**Definition 2.** *The F-F AB integral for 𝒳(t) with M-L kernel is given by*


$${\;}^{ff}{ℐ}_{x_{1},t}^{\tau ,\rho }𝒳(t)=\frac{\rho (1-\tau )t^{\rho -1}}{𝒩(\tau )}𝒳(t)+\frac{\tau \rho }{𝒩(\tau )\Gamma (\tau )}\int_{x_{1}}^{t}(t-s{)}^{\tau -1}s^{\rho -1}𝒳(s)ds.$$

It can be easily understood that by pluging ρ=1, the F-F derivative ${\;}^{ff}{𝒟}_{x_{1},t}^{\tau ,\rho }$ is the standard RL derivative ${\;}^{RL}{𝒟}_{x_{1},t}^{\tau }$ of fractional order τ in AB sense.

**Definition 3.** *The RL derivative in AB sense for the function 𝒳(t) with M-L kernel is given by*


$${\;}^{RL}{𝒟}_{x_{1},t}^{\tau }𝒳(t)=\frac{𝒩(\tau )}{1-\tau }\frac{d}{dt}\int_{x_{1}}^{t}𝒳(s){ℰ}_{\tau }\left[-\frac{\tau }{1-\tau }(t-s{)}^{\tau }\right]ds.$$

A promising avenue with many benefits is the use of F-F operator with double F&F orders in scientific research. This method captures complex patterns and irregularities that traditional methods might miss, allowing for a more sophisticated and strengthened representation of intricate systems by utilizing the two orders. The combination of fractional calculus and fractal geometry allows for more realistic analysis and modeling of real-life events, reflecting the intrinsic identical structures and fractional order dynamics with greater precision. This helps us better understand complex processes and create more reliable mathematical models that are applicable to a wide range of fields. The application of this operator could transform domains as diverse as image analysis to signal processing by providing a flexible toolbox to tackle problems requiring a more profound understanding of complex, multi-fractal behaviors. Accepting this novel paradigm opens up new avenues for research and discovery by promoting a more exact and comprehensive approach in scientific studies. Here, we analyze a COVID-19 fractal–fractional model according to a number of distinct attributes.

**Remark 1.** *For sake of easiness, it is considered that $\frac{𝒩(\tau )}{1-\tau }=P(\tau )$ and $\frac{\tau }{𝒩(\tau )\Gamma (\tau )}=Q(\tau )$.*

## The model: Formulation and assumptions

**Fig 2:**
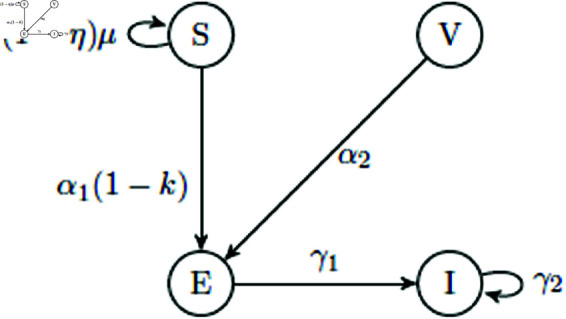
Schematic flow-diagram. Visualization of the model Eq 1.

Motivated with the scientific studies in the previous sections, an F-F mathematical COVID-19 SEVI (*Susceptive-Exposed-Vaccinated-Infected*) model in eco-sociological sense is proposed and is represented by Eq 1. The schematic diagram for which can be seen in Fig 2. The description of the involved parameters or the ratios of transformations are stand for η: vaccination rate, μ: recruitment rate of susceptives, $\alpha_{1}$: infection rate when a susceptive and an infected come into contact due to poor environment, $\alpha_{2}$: infection rate when a vaccinated and an infected come into contact in hygienic surroundings, $\gamma_{1}$: rate coefficient at which exposed individuals develop infection, and $\gamma_{2}$: demise rate of infected individuals. In support of our literature survey, it is possible to consider the rates $\alpha_{1}$, and $\alpha_{2}$. The deficient quality of the environment plays an important role in the infection transmission [60–63], for which the causes may be transmission mechanism (aerosols, surface contamination, etc.), ventilation, surface and hygiene, etc. Now, a mathematical framework for COVID-19 [64] is sought, and consider a parameter *k* called constant probabilistic rate coefficient by which measures other than drugs/vaccines that lower the virus’s infection rate are applied to the epidemic. This calls for an expression for $\alpha_{1}$ w.r.t. aforementioned non-drugs measure. Poor ecosystem in which the infection spread considered is worthy of attention.

The two other parameters: $\alpha_{11}$ and $\alpha_{12}$ as infection rates before and after non-pharmacological interventions, respectively are considered. If *t*_1_ and *t*_2_ be the times spent before and after those interventions, respectively, then $\alpha_{1}=\frac{\alpha_{11}t_{1}+\alpha_{12}t_{2}}{t_{1}+t_{2}}$. Thus, a four-class compartment model is formed as follows:

$${\;}^{ff}{𝒟}_{0,t}^{\tau ,\rho }S(t)=\underset{incoming}{\underbrace{(1-\eta )\mu }}-\underset{exposed}{\underbrace{\alpha_{1}(1-k)IS}},{\;}^{ff}{𝒟}_{0,t}^{\tau ,\rho }E(t)=\alpha_{1}(1-k)IS+\alpha_{2}IV-\underset{infected}{\underbrace{\gamma_{1}E}},{\;}^{ff}{𝒟}_{0,t}^{\tau ,\rho }V(t)=-\underset{exposed}{\underbrace{\alpha_{2}IV}},{\;}^{ff}{𝒟}_{0,t}^{\tau ,\rho }I(t)=\gamma_{1}E-\underset{death}{\underbrace{\gamma_{2}I}},$$ (1)

where the initial conditions are (S(0),E(0),V(0),I(0))≥0. The assumptions considered in this mathematical scheme are stated below as follows:

A1. The compartment *S* contains a set of susceptive individuals who are in danger of getting COVID-19 at the beginning of the model. These individuals enter the framework at a rate of (1−η)μ. It is believed that vaccination provides some defense against the virus.
A2. Neglecting preventive measures such as a obeying government policies, clean habitat, prioritizing earth’s health, etc. other than drugs/vaccines that hightens the infection is given by probabilistic rate constant (1–*k*). So, there will be transfer of infection in virus-exposed compartment *E* from *S* indicated by the term $\alpha_{1}(1-k)IS$.
A3. Individuals got infected after vaccination, thus a transfer term is considered from vaccinated class *V* to *E* given by the term $\alpha_{2}IV$. The susceptive individuals are prone to infection than vaccinated individuals, that is $\alpha_{1}>\alpha_{2}$.
A4. The virus-exposed individuals are not allowed to take pharmacological measures to prevent from the infection, and thus got infected. This term is indicated by $\gamma_{1}E$.
A5. An ecosystem is so considered that only infected individuals will face death, denoted by the term $\gamma_{2}I$.

**Table 1:** The parameter values of model Eq 1 for point of reference. For $\alpha_{1}$, $t_{1}=t_{2}=30$ are taken with suitable values of $\alpha_{11}$ and $\alpha_{12}$ [64].

Parameters:	$\alpha_{1}$	$\alpha_{2}$	k	$\gamma_{1}$	$\gamma_{2}$	η	μ
Values:	$\frac{632}{10^{6}}$	$\left(\frac{3.8}{10^{7}},\frac{1.460}{10^{6}}\right)$	0.6	$\frac{1}{3}$	$\frac{1}{7}$	0.8 (estimated)	100 (estimated)
Units:	${\text{d}}^{-1}{\text{p}}^{-1}$	${\text{d}}^{-1}{\text{p}}^{-1}$	—	${\text{pd}}^{-1}$	${\text{pd}}^{-1}$	—	${\text{pd}}^{-1}$

**Note:** The abbreviations in the “Units" row are as follows: ${\text{d}}^{-1}{\text{p}}^{-1}$ (per day per population), and ${\text{pd}}^{-1}$ (population per day).

With appropriate understanding of research gaps and breaking out the stiffness in classical trend, three main key-features can be indentified as follows:

Kf1. The fractal-fractional derivative used in this study is a special case of the Hattaf F-F operator introduced in [65]. This approach offers a more accurate representation of persistent memory and reliance on disease dynamics. The non-singular kernel is taken as it can portray complex real life phenomena in a comfortable mathematical and numerical analysis that can be treated as a small leap towards understanding biological dynamics, which changes on each passing seconds.
Kf2. The term τ is related to memory effect of our proposed dynamical system. This fractional order τ when approaches towards zero (called long memory) will make the time-dependent kernel vanish. Also, the effect of previous solution will affect the upcoming solution of the dynamical system when τ tends to unity (called short memory) [66]. A disease like COVID-19 is highly sensitive to pharmacological measures, environmental hygiene, etc., which varies very rapidly. Therefore, unlike AIDS, TB, HIV, the dynamical rate of COVID-19 infection can be monitored properly using short memory.
Kf3. This research would establish a SEVI model in order to identify the internal mechanism driven by the virus to create workable and environmentally sound prevention strategies.
Kf4. The article focuses on the impact of subsequent optimal control functions in two classes: susceptive (where awareness control and vaccine control are noted) and vaccinated (where optimal vaccination control is noted). In short, a brief study have been conducted on modeling and optimal control of the COVID-19 SEVI model.

### Biological significance of fractional parameters

In general, relying on the definition of fractional calculus, long-term dependencies and histories are captured in dynamic systems, with this specific reference being known as the memory effect of F-F derivative. Situation with short-term diseases, such as COVID-19, the mentioned concept of the F-F derivative involves its usage depending on the specific disease’s features and the type of processes affecting the infection transmission and development. A short-term disease can also prove to be quite dynamic with interactions and non-local aspects as in the disease spread rates or latent periods or negative feedback in small communities or enclosure. What is more, the F-F derivative can encompass these aspects since it takes into consideration the entire history of the process, although within a shorter time span. For instance, if disease spread depends on the lower past numbers of contacts and exposures, coordinated in the last few days or weeks, fractional derivatives are more relevant to modeling than standard ones. It is then possible to refine the model’s predictions of the future course of the outbreak by including memory effects in the contact patterns and changes in the environment.

The parameters τ: memory effect and ρ: fractional diffusion has biologically significant roles. Here’s a detailed analysis of their significance:

(i) τ
  (a) It includes points like the rate and tenor of interactions between susceptible and infected people. These are socio-political, such as interactions between people and their compliance with prevention measures (such as wearing masks and maintaining physical distance), as well as population density.
  (b) Effective public health interventions aim to reduce its numerical value. Measures such as vaccination, quarantine, and hygiene practices directly target reducing again its numerical value.
(ii) ρ
  (a) It depends on the response of the people’s immunity. Enhancing the immune system is likely to increase the recovery rate while a week immune system is likely to decrease the rate.
  (b) Access to and quality of healthcare significantly affect its numerical value. Effective medical treatments, early detection, and supportive care can increase its numerical value.

This as far as disease transmission is concerned means that τ explains how previous infection and vaccination rates determine the current status of the epidemic; it signifies that the spread of the virus and the response of the population are not abrupt actions of the present but actions in the light of past events and conditions. Fractional diffusion as is explained through ρ shows that the effects of the disease do not decay exponentially but rather follow a more complicated history dependent process. It incorporates the variation in the time-scale of recovery between people and perhaps in the efficacy of medical treatments across time.

The F-F operator, ${\;}^{ff}{𝒟}_{0,t}^{\tau ,\rho }$, can account for the non-local source-dependent and history-dependent transmission and recovery rates of the disease. This is inseparable from biological answers outlined above. Their combined significance can be put together as follows:

(i) *Intervention strategies*: As such, comprehension and correct assessment of τ and ρ are central to learning efficient ways of preventing the condition. Preventive measures will help to lower the parameter τ while medical actions will contribute to raising the parameter ρ controlling the diffusion of the disease.
(ii) *Predictive modeling*: Implementation of τ and ρ improves the model by making it more possible to predict the peaks, the length, and effect of certain public health measures.
(iii) *Resource Allocation*: Evaluations derived from the proposed model can be used to determine the distribution of healthcare support concentrating on regions consisting high transmittance of τ and regions consist low recovery rates ρ to increase its measure of medical care provision.

The research findings in this article is quite important for the sake of synergy between mathematics and nature. The authors have tried to bring a paradigm shift that has never done before in formulating a fractional mathematical model depicting COVID-19 transmission under poor environment hygiene with support of proper literature surveys.

### Model analysis

This section examines and deals in the solution’s qualitative analysis such as existence-uniqueness, non-negativity, and boundedness properties.

#### Existence-uniqueness of solutions.

The development of solutions and the numerical results of the F-F order model (1) are one of the main features of this article, mathematically.

Here, a Banach space is constructed by considering a collection $𝒞=[0,\infty )\times {\mathbb{R}}_{+}^{4}$ and a rule f:𝒞→ℝ defined by $f(S,E,V,I)=\max_{t\in [0,\infty )}\left\{|S|+|E|+|V|+|I|\right\}$. The “fixed point theorem" is imposed to investigate the existence of the solutions for the model. For this, let


$${ℳ}_{1}(S,E,V,I)=(1-\eta )\mu -\alpha_{1}(1-k)IS,$$

$${ℳ}_{2}(S,E,V,I)=\alpha_{1}(1-k)IS+\alpha_{2}IV-\gamma_{1}E,$$

$${ℳ}_{3}(S,E,V,I)=-\alpha_{2}IV,$$

$${ℳ}_{4}(S,E,V,I)=\gamma_{1}E-\gamma_{2}I.$$

In this scenario, the F-F system (1) can be transformed into RL derivative system in AB sense as follows:

$${\;}^{RL}{𝒟}_{0,t}^{\tau }𝒳(t)=\rho t^{\rho -1}𝒴(t,𝒳(t)).$$ (2)

In the above context,


$$𝒳(t)={\left(S(t),E(t),V(t),I(t)\right)}^{T}$$

$$𝒴(t,𝒳(t))={\left({ℳ}_{1},{ℳ}_{2},{ℳ}_{3},{ℳ}_{4}\right)}^{T}.$$

After imposing Definition 3 on Eq 2, it is calculated that

$$P(\tau )\frac{d}{dt}\int_{0}^{t}𝒴(s,𝒳(s)){ℰ}_{\tau }\left[-\frac{\tau }{1-\tau }(t-s{)}^{\tau }\right]ds=\rho t^{\rho -1}𝒴(t,𝒳(t)).$$ (3)

Now, help of Definition 2 in Eq 3 has been taken to get


$$𝒴(t)=𝒴(0)+\frac{\rho t^{\rho -1}𝒴(t,𝒳(t))}{P(\tau )}+\rho Q(\tau )\int_{0}^{t}(t-s{)}^{\tau -1}𝒴(s,𝒳(s))s^{\rho -1}ds.$$

An analysis present in [46] is sought, and then a set ${𝒜}_{x_{1}}^{x_{2}}={𝒢}_{m}(t_{m})\times {𝒱}_{0}(t_{0})$ is considered, where ${𝒢}_{m}(t_{m})=[t_{m}-x_{1},t_{m}+x_{1}]$ and ${𝒱}_{0}(t_{0})=[t_{0}-x_{2},t_{0}+x_{2}]$. Suppose $𝒵=\sup_{t\in {𝒜}_{x_{1}}^{x_{2}}}f(𝒴)$, and the norm *f* is defined by $f_{\infty }(l)=\sup_{t\in {𝒜}_{x_{1}}^{x_{2}}}|l(t)|$. Keeping forward the mathematical sentiment, a Banach contraction map $n:ℋ({𝒢}_{m}(t_{m}),{𝒱}_{x_{2}}(t_{m}))\to ℋ({𝒢}_{m}(x_{2}),{𝒱}_{x_{2}}(t_{m}))$ defined by


$$n𝒳(t)={𝒳}_{0}+\frac{\rho t^{\rho -1}𝒴(t,𝒳(t))}{P(\tau )}+\rho Q(\tau )\int_{0}^{t}(t-s{)}^{\tau -1}𝒴(s,𝒳(s))s^{\rho -1}ds$$

is considered. At this stage, it is needed to show $f(n𝒳(t)-{𝒳}_{0})<w$ in a such a way that


$$f(n𝒳(t)-{𝒳}_{0})\leq \frac{\rho t^{\rho -1}f(𝒴(t,𝒳(t)))}{P(\tau )}+\rho Q(\tau )\int_{0}^{t}(t-s{)}^{\tau -1}f(𝒴(s,𝒳(s)))s^{\rho -1}ds,$$

$$\leq \frac{\rho t^{\rho -1}𝒵}{P(\tau )}+\rho Q(\tau )𝒵\int_{0}^{t}(t-s{)}^{\tau -1}s^{\rho -1}ds.$$ (4)

Let *s* = *ut* in Ineq 4, which implies


$$f(n𝒳(t)-{𝒳}_{0})\leq \frac{\rho t^{\rho -1}𝒵}{P(\tau )}+\rho Q(\tau )𝒵s^{\rho +\tau }ℛ(\rho ,\tau ).$$

This is reduced to


$$f(n𝒳(t)-{𝒳}_{0})<w\to 𝒵<\frac{wℛ(\rho ,\tau )𝒩(\tau )\Gamma (\tau )}{(1-\tau )\Gamma (\tau )\rho t^{\rho -1}+\tau \rho t^{\rho +\tau }}.$$

Let, ${𝒳}_{1},{𝒳}_{2}\in ℋ({𝒢}_{m}(t_{m}),{𝒱}_{x_{2}}(t_{m}))$. Therefore,


$$f(n{𝒳}_{1}-n{𝒳}_{2})\leq \frac{\rho t^{\rho -1}f(𝒴(t,{𝒳}_{1}(t))-𝒴(t,{𝒳}_{2}(t)))}{P(\tau )}\;+\;\rho Q(\tau )\int_{0}^{t}(t-s{)}^{\tau -1}f(𝒴(s,{𝒳}_{1}(s))-𝒴(s,{𝒳}_{2}(s)))s^{\rho -1}ds.$$

By the definition of the map *n*, it can be written that


$$f(n{𝒳}_{1}-n{𝒳}_{2})\leq \frac{\rho t^{\rho -1}𝒴(t)f_{\infty }({𝒳}_{1}(t)-{𝒳}_{2}(t))}{P(\tau )}\;+\;\rho Q(\tau )𝒴(t)\int_{0}^{t}(t-s{)}^{\tau -1}f_{\infty }({𝒳}_{1}(s)-{𝒳}_{2}(s))s^{\rho -1}ds\;\leq \frac{\rho t^{\rho -1}𝒴f_{\infty }({𝒳}_{1}-{𝒳}_{2})}{P(\tau )}+\rho Q(\tau )𝒴f_{\infty }({𝒳}_{1}-{𝒳}_{2})t^{\rho +\tau }ℛ(\rho ,\tau ).\;f(n{𝒳}_{1}-n{𝒳}_{2})\leq \left[\frac{\rho t^{\rho -1}𝒴}{P(\tau )}+\rho Q(\tau )𝒴t^{\rho +\tau }ℛ(\rho ,\tau )\right]f_{\infty }({𝒳}_{1}-{𝒳}_{2}).$$

It can be strongly said that if $f(n{𝒳}_{1}-n{𝒳}_{2})\leq f_{\infty }({𝒳}_{1}-{𝒳}_{2})$, then the function *n* is a Banach Contraction map. This evaluate to the following two inequalities:


$$1<{\left(\frac{\rho t^{\rho -1}𝒴}{P(\tau )}+\rho Q(\tau )𝒴t^{\rho +\tau }ℛ(\rho ,\tau )\right)}^{-1},$$

and


$$𝒵<{\left(\frac{\rho t^{\rho -1}𝒴}{P(\tau )}+\rho Q(\tau )𝒴t^{\rho +\tau }ℛ(\rho ,\tau )\right)}^{-1},$$

which fulfills every requirement for the proposed F-F order COVID-19 system’s unique solutions.

#### Non-negativity and Boundedness of solutions.

A theorem is provided to glance upon this property pondering a hyperplane [67].

**Theorem 1.** *Solutions of the proposed model (1), which initiate at ${\mathbb{R}}_{+}^{4}$, are non-negative and bounded uniformly.*

**Proof 1** (Proof). *From the model (1), it can be seen that*

$${\;}^{ff}{𝒟}_{0,t}^{\tau ,\rho }S(t){|}_{S=0}=(1-\eta )\mu \;>0,{\;}^{ff}{𝒟}_{0,t}^{\tau ,\rho }E(t){|}_{E=0}=\alpha_{1}(1-k)IS+\alpha_{2}IV\;\geq 0,{\;}^{ff}{𝒟}_{0,t}^{\tau ,\rho }V(t){|}_{V=0}=0,{\;}^{ff}{𝒟}_{0,t}^{\tau ,\rho }I(t){|}_{I=0}=\gamma_{1}E\;\geq 0.$$ (5)

*As per this, if $\left(S(0),E(0),V(0),I(0)\right)\in {\mathbb{R}}_{+}^{4}$, the solution cannot run away from the hyperplane. Also, the collection of points of vector field into ${\mathbb{R}}_{+}^{4}$ is a non-negative invariant set captivating all orthant on each hyperplane.*

### Vaccine-clearance equilibrium point

In this part, the only equilibrium point of model (1) is assessed, and sketched a biological interpretation.

A region $ℬ=\left\{\left(S,E,V,I\right)\in {\mathbb{R}}_{+}^{4}\cup \left\{0\right\}:\left(S(0),E(0),V(0),I(0)\right)\geq 0\right\}$ is defined. A biological significance limit also applies to each state variable in this case. That’s why the region ℬ as a whole is called a “Global" domain. As of right now, model (1)’s equilibrium point is given by solving the set of following non-linear equations:


$$(1-\eta )\mu -\alpha_{1}(1-k)IS=0,$$

$$\alpha_{1}(1-k)IS+\alpha_{2}IV-\gamma_{1}E=0,$$

$$-\alpha_{2}IV=0,$$

$$\gamma_{1}E-\gamma_{2}I=0.$$

Thus, the only equilibrium point is ${ℬ}_{0}=\left(\frac{\gamma_{2}}{\alpha_{1}(1-k)},\frac{(1-\eta )\mu }{\gamma_{1}},0,\frac{(1-\eta )\mu }{\gamma_{2}}\right)$.

This means that the infection co-exist in the environment, and it will persists in our environment like other epidemics such as HIV, TB, Malaria, Dengue, etc.[68–71].

## The basic and the effective reproduction number

The basic reproduction number may be defined as the ratio, denoted by *R*_0_, of the fresh seronegative persons in a population who acquire it from a sick person in the population to the size of the said population. Equating the R.H.S of the fourth equation of model (1) to zero gives $\frac{E}{I}=\frac{\gamma_{2}}{\gamma_{1}}$. This ratio is *R*_0_. In general, there are two broad epidemiological description for *R*_0_. Infection probably tends to decrease over time if *R*_0_<1 because the virus will become dormant. However, in the event that *R*_0_>1, the infection is likely to proliferate and consequently spread quickly.

We incorporate “Next Generation Matrix" method to evaluate the effective reproduction rate, *R*_*t*_. The compartments that are responsible for infection transmission given by the model (1) are virus-exposed individuals (*E*) and infected individuals (*I*). Their F-F rate of change is given the following set of differential equations:


$${\;}^{ff}{𝒟}_{0,t}^{\tau ,\rho }E(t)=\alpha_{1}\left(1-k\right)IS+\alpha_{2}IV-\gamma_{1}E,$$

$${\;}^{ff}{𝒟}_{0,t}^{\tau ,\rho }I(t)=\gamma_{1}E-\gamma_{2}I\;=\;0-\left(-\gamma_{1}E+\gamma_{2}I\right).$$

Let, the matrix of new infections be denoted and given by $𝔽=(\begin{array}{c} \alpha_{1}(1-k)IS+\alpha_{2}IV\\ 0 \end{array})$ and matrix of transitions be denoted and given by $𝕍=(\begin{array}{c} \gamma_{1}E\\ -\gamma_{1}E+\gamma_{2}I \end{array})$. Let, their linearized versions be denoted and given by $𝔉=(\begin{array}{cc} 0 & \alpha_{1}(1-k)S+\alpha_{2}V\\ 0 & 0 \end{array})$ and $𝔙=(\begin{array}{cc} \gamma_{1} & 0\\ -\gamma_{1} & \gamma_{2} \end{array})$, respectively. The matrix multiplication of matrices 𝔉 and ${𝔙}^{-1}$ is $𝔉{𝔙}^{-1}=(\begin{array}{cc} \frac{\alpha_{1}(1-k)S+\alpha_{2}V}{\gamma_{2}} & \frac{\alpha_{1}(1-k)S+\alpha_{2}V}{\gamma_{2}}\\ 0 & 0 \end{array})$. We now impose initial condition of model (1) on $𝔉{𝔙}^{-1}$ and find the the largest eigenvalue for $𝔉{𝔙}^{-1}(S(0),E(0),V(0),I(0))$ [64]. The term $\frac{\alpha_{1}(1-k)S(0)+\alpha_{2}V(0)}{\gamma_{2}}$ is its spectral radius, which is called the effective reproduction number and is denoted by *R*_*t*_.

**Fig 3:**
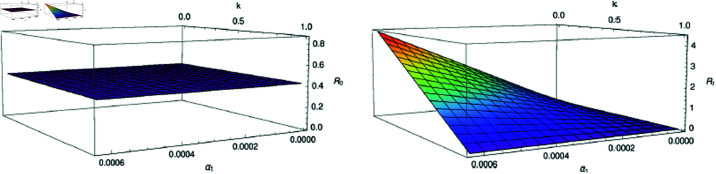
Visualization of reproduction numbers in terms of infection rate due to unhygienic environment and non-pharmacological measure.

The spectral radius of $𝔉{𝔙}^{-1}({ℬ}_{0})$ is unity i.e., $R_{t}({ℬ}_{0})=1$. As discussed in subsection “Model analysis", this case suggests about co-existence of the illness converting the epidemic to endemic. Infection strictly tends to decrease over time if *R*_*t*_<1 because the virus will become dormant. However, in the case that *R*_*t*_>1, the infection strongly proliferate and spread very quickly. The conceptual difference between the reproductive numbers is that *R*_0_ deals epidemic in probabilistic sense, whilst *R*_*t*_ does the same deterministically. Since population modeling is dynamic, thus *R*_*t*_ is much important than *R*_0_. The number *R*_*t*_ describe about the present condition of the susceptives that can be changed due to vaccination. Understanding *R*_*t*_ helps in crucial real-time for decisions to make during the infection transmission. This allows the government to issue strategies or adjust plans on the basis of current scenario. Thus, the parameters $\alpha_{1}$ and *k* are very much significant in the context of infection transmission and environment hygiene as shown in Fig 3.

**Theorem 2.** *The vaccine-clearance equilibrium point ${ℬ}_{0}$ of the model (1) with parameters $\left(\eta ,\mu ,\alpha_{1},\alpha_{2},\gamma_{1},\gamma_{2}\right)$ is stable.*

**Proof 2** (Proof). *At ${ℬ}_{0}$, the determinantal polynomial in variable w is given by $c(w)=w^{4}+\Delta_{3}w^{3}+\Delta_{2}w^{2}+\Delta_{1}w+\Delta_{0}$, where the co-efficients $\Delta_{\phi =0,1,2,3}$ are as follows:*

$\Delta_{0}$: $\left(1-k\right)\left(1-\eta \right){\;}^{2}\mu^{2}\alpha_{1}\alpha_{2}\gamma_{1}\gamma_{2}^{-1}\;>\;0$,
$\Delta_{1}$: $\left(1-k\right)\mu \alpha_{1}\left(\gamma_{1}+\gamma_{2}\right)\left\{\left(1-\eta \right){\;}^{2}\mu \alpha_{2}+\gamma_{1}\gamma_{2}\right\}\gamma_{2}^{-2}\;>\;0$,
$\Delta_{2}$: $\left(1-\eta \right)\mu \left[\left(1-k\right)\alpha_{1}\left\{\left(1-\eta \right)\mu \alpha_{2}+\gamma_{1}\gamma_{2}+\gamma_{2}^{2}+\alpha_{2}\gamma_{2}\left(\gamma_{1}+\gamma_{2}\right)\right\}\right]\gamma_{2}^{-2}\;>\;0$,
$\Delta_{3}$: $\left[\left(1-\eta \right)\mu \left\{\left(1-k\right)\alpha_{1}+\alpha_{2}\right\}+\gamma_{2}\left(\gamma_{1}+\gamma_{2}\right)\right]\gamma_{2}^{-1}\;>\;0$.

*The values of the parameters involved are always positive due to their biological significance. In total, the argument implies the following:*

(i) $\Delta_{\phi =0,1,2,3}>0$,
(ii) the polynomial *c*(*w*) has no positive real roots,
(iii) according to Descarte’s rule of sign, replacing *w* by –*w* in *c*(*w*) indicates that the polynomial *c*(*w*) has 4 negative roots.

*Therefore, the determinantal equation c(w)=0 gives 4 negative eigenvalues implying stablity of the equilibrium point ${ℬ}_{0}$.*

## Sensitivity analysis

Sensitivity analysis sheds light on how important each parameter is in the disease dynamics with regard to the infection spread. High sensitivity parameters need to be handled carefully because even a small change in them can have a significant quantitative impact on *R*_0_ and *R*_*t*_.

The goal is to identify the most important variables linked to particular intervention that significantly affects the dynamics of disease. The parameters are ought to look at that lead to a significant variation in the *R*_0_’s and *R*_*t*_’s values. To ascertain the related changes in the state variables brought about by a change in a parameter, one can utilize the sensitivity index (denoted by s.i) [72]. The “normalized forward sensitivity indices" (NFSI) [73] of *R*_0_ and *R*_*t*_ is sought. Let, *c* be any figurative portrayal of parameters found in the *R*_0_ and *R*_*t*_ expressions. Therefore, NFSI for *R*_0_ and *R*_*t*_ are repectively calculated and formulated as;

$${𝔖}_{R_{0}}^{c}=\frac{\partial R_{0}}{\partial c}\times \frac{c}{R_{0}}\;{𝔖}_{R_{t}}^{c}=\frac{\partial R_{t}}{\partial c}\times \frac{c}{R_{t}}.$$ (6)

**Fig 4:**
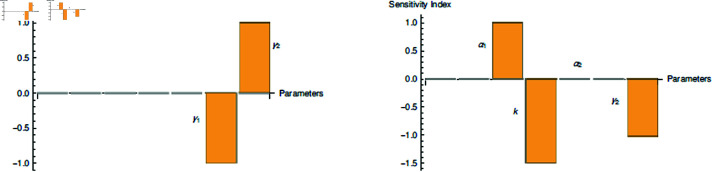
Visualization of (left) ${𝔖}_{R_{0}}^{c}$ and (right) ${𝔖}_{R_{t}}^{c}$ along vertical axis, where c represents significant parameters labelled already. We have taken $\alpha_{2}=10^{-7}$, which implies ${𝔖}_{R_{t}}^{\alpha_{2}}>0$ (see Table 2). The other relevant parameters are taken from Table 1.

The sign of s.i gives a varying relationship between the reproduction number and associated parameter. The parameter’s influence will be greater if s.i has magnitude of larger value. The explanation of the Table 2 together with Fig 4 can be re-written as follows:

E1. An increment or decrement of 1% in $\gamma_{1}$ always decreases the value of *R*_0_ by 1%.
E2. An increment (or decrement) in value of $\gamma_{2}$ by 1% will increase (or decrease) the value of *R*_0_ by 1%.
  But, an increment or decrement of 1% in $\gamma_{2}$ always decreases the value of *R*_*t*_ by 1%.
E3. An increment (or decrement) of 1% in $\alpha_{1}$ increases (or decreases) the value of *R*_*t*_ almost by 1%.
E4. An increment (or decrement) of 1% in $\alpha_{2}$ definitely has a positive impact in the value of *R*_*t*_ but very negligible.
E5. An increment or decrement of 1% in *k* always tremendously decreases the value of *R*_*t*_ by 1.5%.

**Table 2:** Description of the sensitivity indices and behaviour of parameters involved in $R_{0}$ and $R_{t}$.

s.i:	${𝔖}_{R_{0}}^{\gamma_{1}}$	${𝔖}_{R_{0}}^{\gamma_{2}}$	${𝔖}_{R_{t}}^{\alpha_{1}}$	${𝔖}_{R_{t}}^{\alpha_{2}}$	${𝔖}_{R_{t}}^{k}$	${𝔖}_{R_{t}}^{\gamma_{2}}$
Value:	–1	+ 1	+ 0.99	$1.92\times 10^{-8}$	–1.5	–1
Nature of impact:	–ve	+ve	+ve	+ve	–ve	–ve

Apart from the parameters discussed in (E1)–(E5), the other parameters of model (1) has null effect on *R*_0_ and *R*_*t*_.

## U-H stability

In this section, U-H stability for the COVID-19 SEVI model (1) is investigated. An important definition has to be seek, which is mentioned below [46]:

**Definition 4.** *The model (1) is said to be U-H stable if for each (𝚂,𝙴,𝚅,𝙸)∈ℬ there exists $h_{\phi }>0$ such that a real number ${𝒲}_{\phi }>0$ is defined satisfying the set of inequalities*

$${|}^{ff}{𝒟}_{0,t}^{\tau ,\rho }𝚂(t)-{ℳ}_{1}(𝚂,t)|\leq {𝒲}_{1},\;{|}^{ff}{𝒟}_{0,t}^{\tau ,\rho }𝙴(t)-{ℳ}_{2}(𝙴,t)|\leq {𝒲}_{2},\;{|}^{ff}{𝒟}_{0,t}^{\tau ,\rho }𝚅(t)-{ℳ}_{3}(𝚅,t)|\leq {𝒲}_{3},\;{|}^{ff}{𝒟}_{0,t}^{\tau ,\rho }𝙸(t)-{ℳ}_{4}(𝙸,t)|\leq {𝒲}_{4},$$ (7)

*where ϕ=1,2,3,4. Also, for any time t there exists (𝒮(t),ℰ(t),𝒱(t),ℐ(t)) such that*


$$f\left(𝒮(t)-𝚂\right)\leq h_{1}{𝒲}_{1},$$

$$f\left(ℰ(t)-𝙴\right)\leq h_{2}{𝒲}_{2},$$

$$f\left(𝒱(t)-𝚅\right)\leq h_{3}{𝒲}_{3},$$

$$f\left(ℐ(t)-𝙸\right)\leq h_{4}{𝒲}_{4}.$$

Here, ${ℳ}_{\phi }$ and rule *f* are same as discussed in subsection “Existence-uniqueness of solutions".

**Remark 2.** 𝚂 be a solution of the first inequality present in system (7) if and only if there exists smooth curve ${𝒜}_{1}$ such that

i. $|{𝒜}_{1}|<{𝒲}_{1}$,
ii. ${\;}^{ff}{𝒟}_{0,t}^{\tau ,\rho }𝚂(t)={ℳ}_{1}+{𝒜}_{1}$.

In the same manner, appropriate smooth curves for the state variables 𝙴, 𝚅, and 𝙸 can be found.

**Theorem 3.** *The model (1) is U-H stable if*

$$\delta_{\phi }\left[\frac{\rho Q(\tau ){\left\{\Gamma (\tau )\right\}}^{2}}{\Gamma (\rho +\tau )}+\frac{\rho }{P(\tau )}\right]\leq 1,$$ (8)

*where ϕ=1,2,3,4 and corresponding $\delta_{\phi }$ are functions of $h_{\phi }$ respectively assigned for S, E, V and I.*

**Proof 3** (Proof). *Let, 𝚂(t) and ${𝒲}_{1}$ be both positive. Therefore, ${|}^{ff}{𝒟}_{0,t}^{\tau ,\rho }𝚂(t)-{ℳ}_{1}(𝚂,t)|\leq {𝒲}_{1}$. Using the Remark 2(ii), it can be followed that*

$${\;}^{ff}{𝒟}_{0,t}^{\tau ,\rho }𝚂(t)={ℳ}_{1}+{𝒜}_{1}.$$ (9)

*Employing AB integral (see Definition 2) to the Eq* 9 *gives*


$$𝚂(t)-S(0)=\frac{\rho }{Q(\tau )}\int_{0}^{t}{\left(t-l\right)}^{\tau -1}l^{\rho -1}{ℳ}_{1}\left(l,𝚂(l)\right)dl+\frac{\rho }{P(\tau )}t^{\rho -1}{ℳ}_{1}\left(l,𝚂(t)\right)\;+\;\frac{\rho }{Q(\tau )}\int_{0}^{t}{\left(t-l\right)}^{\tau -1}l^{\rho -1}{𝒜}_{1}(l)dl+\frac{\rho }{P(\tau )}t^{\rho -1}{𝒜}_{1}.$$

*Suppose 𝒮 is a distinct solution for the model (1). This implies,*


$$𝒮(t)-S(0)=\frac{\rho }{Q(\tau )}\int_{0}^{t}{\left(t-l\right)}^{\tau -1}l^{\rho -1}{ℳ}_{1}\left(l,𝒮(l)\right)dl+\frac{\rho }{P(\tau )}t^{\rho -1}{ℳ}_{1}\left(l,𝒮(t)\right)$$

*This implies that*


$$f\left(𝚂(t)-𝒮(t)\right)\leq \frac{\rho }{Q(\tau )}\int_{0}^{t}{\left(t-l\right)}^{\tau -1}l^{\rho -1}|{ℳ}_{1}\left(l,𝚂(l)\right)-{ℳ}_{1}\left(l,𝒮(l)\right)dl|\;+\;\frac{\rho }{P(\tau )}t^{\rho -1}|{ℳ}_{1}\left(l,𝚂(l)\right)-{ℳ}_{1}\left(l,𝒮(l)\right)dl|+\frac{\rho }{P(\tau )}t^{\rho -1}|{𝒜}_{1}|\;+\;\frac{\rho }{Q(\tau )}\int_{0}^{t}{\left(t-l\right)}^{\tau -1}l^{\rho -1}|{𝒜}_{1}(l)|dl,\;\leq \delta_{\phi }\left[\frac{\rho Q(\tau ){\left\{\Gamma (\tau )\right\}}^{2}}{\Gamma (\rho +\tau )}+\frac{\rho }{P(\tau )}\right]f\left(𝚂(t)-𝒮(t)\right)\;+\;\left[\frac{\rho Q(\tau ){\left\{\Gamma (\tau )\right\}}^{2}}{\Gamma (\rho +\tau )}+\frac{\rho }{P(\tau )}\right]{𝒲}_{1}.$$

*Arranging further, it can be found that*


$$f\left(𝚂(t)-𝒮(t)\right)\leq \frac{\left[\frac{\rho Q(\tau ){\left\{\Gamma (\tau )\right\}}^{2}}{\Gamma (\rho +\tau )}+\frac{\rho }{P(\tau )}\right]{𝒲}_{1}}{1-\delta_{\phi }\left[\frac{\rho Q(\tau ){\left\{\Gamma (\tau )\right\}}^{2}}{\Gamma (\rho +\tau )}+\frac{\rho }{P(\tau )}\right]}.$$

*Upon the consideration of*


$$h_{1}\leq \frac{\left[\frac{\rho Q(\tau ){\left\{\Gamma (\tau )\right\}}^{2}}{\Gamma (\rho +\tau )}+\frac{\rho }{P(\tau )}\right]}{1-\delta_{\phi }\left[\frac{\rho Q(\tau ){\left\{\Gamma (\tau )\right\}}^{2}}{\Gamma (\rho +\tau )}+\frac{\rho }{P(\tau )}\right]}$$

*yields $f\left(𝚂(t)-𝒮(t)\right)\leq h_{1}{𝒲}_{1}$. In the same manner, it is possible to have $h_{\phi =2,3,4}$ such that for the remaining variables the expressions $f\left(𝙴(t)-ℰ(t)\right)\leq h_{2}{𝒲}_{2}$, $f\left(𝚅(t)-𝒱(t)\right)\leq h_{3}{𝒲}_{3}$ and $f\left(𝙸(t)-ℐ(t)\right)\leq h_{4}{𝒲}_{4}$ are true. Thus, it can be said that model (1) is U-H stable.*

**Remark 3.** *The U-H stability gives a picture of solution robustness with very precise approximate solution, where as the after math of asymptotic stability is about convergence of solution to the equilibrium state.*

## Optimal control strategies

The lock-downs worsened sustainable economic growth whilst having no discernible impact on the decline in COVID-19 fatalities. Public health and containment alone won’t be enough in an instant reaction to the pandemic to protect people’s well-being. The protection of families, individuals, and their means of subsistence against the pandemic’s immediate effects on incomes and access to basic services, as well as the preparation of longer-term domestic resilience and more durable essential services, are all necessary steps in broadening the protection and nurturing of human capital. A study have shown that in the absence of such policy strategies to safeguard citizens and provide basic services, particularly to the most disadvantaged, the development of human capital may be disrupted during shocks [74]. The consequences of this disruption may extend across generations and decades, thereby impairing future welfare and productivity. In addition, equitable financial incentives must be provided in tandem with the“green revival" following the epidemic, conservation of ecosystems, and climate action. Another study have disclosed that depsite saving thousands lives, COVID-19 vaccination campaign costs billions of U.S. currency [75].

### Formation of the control problem

Here, a detail presentation on formulating the optimal control functions and their brief analysis are presented. The two time-dependent control functions $\nu_{1}(t)$: COVID-19 awareness-promotion and $\nu_{2}(t)$: vaccination drive are introduced. The system of control dynamics in F-F sense is given by the model (10), depicted as follows:

$${\;}^{ff}{𝒟}_{0,t}^{\tau ,\rho }S(t)=(1-\eta )\mu -\alpha_{1}(1-k)IS-b_{1}\nu_{1}(t)S+b_{2}\nu_{2}(t)V,{\;}^{ff}{𝒟}_{0,t}^{\tau ,\rho }E(t)=\alpha_{1}(1-k)IS+\alpha_{2}IV-\gamma_{1}E,{\;}^{ff}{𝒟}_{0,t}^{\tau ,\rho }V(t)=-\alpha_{2}IV-b_{2}\nu_{2}(t)V,{\;}^{ff}{𝒟}_{0,t}^{\tau ,\rho }I(t)=\gamma_{1}E-\gamma_{2}I.$$ (10)

**Fig 5:**
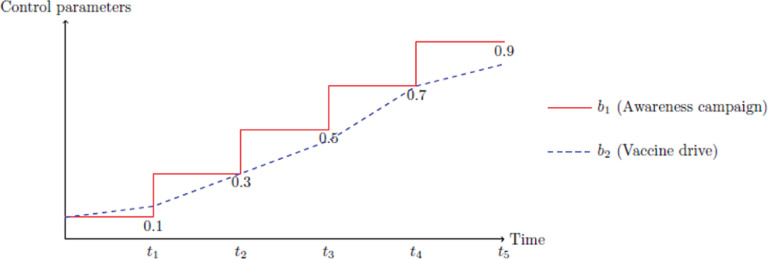
Variation of the control parameters over time.

The initial conditions are same as that of model (1) together with $\nu_{\phi =1,2}(t)=0$. Two control parameters *b*_1_: measures the awareness campaign and *b*_2_: efforts of drug/vaccine efficacy, are incorporated. Empirically, typical values of $b_{\phi =1,2}$ can be taken from the closed interval [0,1]. Lower the values of *b*_1_ and *b*_2_ lenient or weak is the awareness program on COVID-19 and vccination drive, respectively and is represented in Fig 5.

Awareness campaigns are not frequently implemented in continuous way rather often relaxed. This is not for the case of vaccination drive as it improves gradually, hence a smooth curve.

The scheme is to optimize *S* and *V* and simultaneously to minimize the economic cost associated with the two controls $b_{\phi =1,2}$. The economic overhead of optimizing *S* and *V* are represented by the terms *A*_1_*S* and $A_{2}V$, respectively. In the same manner, economic overhead for the control $\nu_{1}$ is given by $\frac{1}{2}B_{1}\nu_{1}^{2}$ and that of $\nu_{2}$ is $\frac{1}{2}B_{2}\nu_{2}^{2}$. Therefore, the objective function is given as follows:

$$\min\;J\left(\nu_{\phi =1,2}\right)=\int_{0}^{t_{end}}(A_{1}S+A_{2}V+\frac{1}{2}\sum_{\phi =1}^{2}B_{\phi }\nu_{\phi }^{2})dt.$$ (11)

There is a need to have optimal solutions $\nu_{\phi =1,2}^{*}$ in such a way that

$$J\left(\nu_{\phi =1,2}^{*}\right)=\min_{\nu_{\phi =1,2}}\left\{J\left(\nu_{\phi =1,2}\right)|\nu_{\phi =1,2}\in ℧\right\},$$ (12)

where ℧ is called control-set defined as $℧=\left\{\left(\nu_{1},\nu_{2}\right)|\nu_{\phi =1,2}:\left[0,t_{end}\right]\to \left[0,\infty \right)\right\}$. Further, each $\nu_{\phi }$ is Lebesgue measurable. The celebrated Pontryagin’s Maximum Principle (PMP) can be implemented to fulfill the sufficient conditions of the optimal control problem. The Eqs 10 and 12 are converted into a minimizing problem in point-wise using a Hamiltonian function $ℋ\equiv ℋ(\nu_{\phi =1,2})$ mentioned below as:

$$ℋ(\nu_{\phi =1,2})=A_{1}S+A_{2}V+\frac{1}{2}\sum_{\phi =1}^{2}B_{\phi }\nu_{\phi }^{2}+\lambda_{S}.^{ff}{𝒟}_{0,t}^{\tau ,\rho }S(t)+\lambda_{E}.^{ff}{𝒟}_{0,t}^{\tau ,\rho }E(t)\;\;+\;\lambda_{V}.^{ff}{𝒟}_{0,t}^{\tau ,\rho }V(t)+\lambda_{I}.^{ff}{𝒟}_{0,t}^{\tau ,\rho }I(t),$$ (13)

where $\lambda_{S}$, $\lambda_{E}$, $\lambda_{V}$ and $\lambda_{I}$ are co-state variables. Their rate of change are given as follows:

$$-\frac{d\lambda_{S}}{dt}\;=\;\frac{\partial ℋ}{\partial S}=A_{1}+\lambda_{S}\left\{-\alpha_{1}(1-k)I-b_{1}\nu_{1}\right\}+\lambda_{E}\left\{\alpha_{1}(1-k)I\right\},\;-\frac{d\lambda_{E}}{dt}\;=\;\frac{\partial ℋ}{\partial E}=\lambda_{E}\left(-\gamma_{1}\right)+\lambda_{I}\left(\gamma_{1}\right),\;-\frac{d\lambda_{V}}{dt}\;=\;\frac{\partial ℋ}{\partial V}=A_{2}+\lambda_{S}\left(b_{2}\nu_{2}\right)+\lambda_{E}\left(\alpha_{2}I\right)+\lambda_{V}\left(-\alpha_{2}I-b_{2}\nu_{2}\right),\;-\frac{d\lambda_{I}}{dt}=\;\frac{\partial ℋ}{\partial I}=\lambda_{S}\left\{-\alpha_{1}(1-k)S\right\}+\lambda_{E}\left\{\alpha_{1}(1-k)S+\alpha_{2}V\right\}+\lambda_{V}\left(-\alpha_{2}V\right)\;\;+\;\lambda_{I}\left(-\gamma_{2}\right),$$ (14)

where the conditions of transversality are $\lambda_{\phi =S,E,V,I}\left(t_{end}\right)=0$ for $0<\nu_{\phi =1,2}<1$. To get the optimal values of $\nu_{\phi =1,2}^{*}$, mentioned below Eq 15 is sought:

$$\frac{\partial ℋ}{\partial \nu_{1}}=B_{1}\nu_{1}-\lambda_{S}b_{1}S\;=\;0,\;\frac{\partial ℋ}{\partial \nu_{2}}=B_{2}\nu_{2}+\lambda_{S}b_{2}V-\lambda_{V}b_{2}V\;=\;0.$$ (15)

This implies that

$$\nu_{1}=S\frac{\lambda_{S}b_{1}}{B_{1}},\;\nu_{2}=V\frac{\left(\lambda_{V}-\lambda_{S}\right)b_{2}}{B_{2}}.$$ (16)

### Existence of the controls

An optimal control strategy may said to be well-posed if its solution exists. In this section, a theorem for the control problem existence is posed. The theorem is as follows:

**Theorem 4** [76] *The controls $\nu_{\phi =1,2}^{*}$ that can respectively minimize $\nu_{\phi =1,2}$ over ℧ are given by*

$$\nu_{1}^{*}=\max\left\{\min\left[S\frac{\lambda_{S}b_{1}}{B_{1}},1\right],0\right\},\;\nu_{2}^{*}=\max\left\{\min\left[V\frac{\left(\lambda_{V}-\lambda_{S}\right)b_{2}}{B_{2}},1\right],0\right\},$$ (17)

*where $\lambda_{\phi =S,E,V,I}$ satisfy Eqs* 10*–*17, *$\lambda_{\phi =S,E,V,I}\left(t_{end}\right)=0$, and Eq* 18 *mentioned below as:*

$$\nu_{1}^{*}=\left\{ \begin{array}{c} 0;\;\nu_{1}\leq 0,\\ \nu_{1};\;0<\nu_{1}<1,\\ 1;\;\nu_{1}\geq 1, \end{array}\right.\;\nu_{2}^{*}=\left\{ \begin{array}{c} 0;\;\nu_{2}\leq 0,\\ \nu_{2};\;0<\nu_{2}<1,\\ 1;\;\nu_{2}\geq 1, \end{array}\right..$$ (18)

**Proof 4** (Proof). *The attributes that are to be used to prove the optimal economic-control solution’s existence are (i) convexity property of the function J for the state variables’s solution boundedness, and (ii) Lipschitz’s property of the state’s system concerned to the state variables. The PMP is induced to get the mentioned set of equations as follows: ${\;}^{ff}{𝒟}_{0,t}^{\tau ,\rho }S(t)=\frac{\partial ℋ}{\partial S}$, ${\;}^{ff}{𝒟}_{0,t}^{\tau ,\rho }E(t)=\frac{\partial ℋ}{\partial E}$, ${\;}^{ff}{𝒟}_{0,t}^{\tau ,\rho }V(t)=\frac{\partial ℋ}{\partial V}$, and ${\;}^{ff}{𝒟}_{0,t}^{\tau ,\rho }I(t)=\frac{\partial ℋ}{\partial I}$ with $\lambda_{\phi =S,E,V,I}\left(t_{end}\right)=0$. The optimality conditions can be extracted from Eq* 15.

*The solution of model (10) gives the adjoint systems (13) and (14), and Eq* 17 *gives the optimal controls (16). The controlled system (10) and its initial conditions, the adjoint system (13), and $\lambda_{\phi =S,E,V,I}\left(t_{end}\right)=0$ comprises the optimal system.*

### Numerical explantion of the controls and efficiency index

Mathematically, the optimal control strategy is fragmented into two sub-strategies follows as:

O1. $\left(\nu_{\phi =1,2}\neq 0\right)$: All the controls are present in the system, i.e. their numerical values are not zero.
O2. $\left(\nu_{1}=0,\nu_{2}\neq 0\right)$: Only the vaccination drive control is present in the system and with passage of time awareness campaign cease to exist. The vaccination drive control is always considered to be non-zero for keeping the infection long-time away from the susceptives.

The two strategies in terms of their efficiency is now differentiated. The formula for evaluating efficiency index is denoted and defined by


$$e.i=\left(1-\frac{i^{c}}{i^{0}}\right)\times 100,$$

where ${\text{i}}^{\text{c}}$, ${\text{i}}^{0}$ are cumulative virus-exposed and infected individuals with and without controls, respectively. By using Weedle’s rule of integration and letting the integral $i=\int_{0}^{1}\left\{E(t)+I(t)\right\}dt$, the *e*.*i* is calculated.

It is found that *i*^0^ = 1.26080401033441 and other calculations in detail can be seen in Table 3, which suggests that strategy O1 is effective than that of O2. Implementation of two controls can be costly and economic planning must be considered.

**Table 3:** Efficiency indices values w.r.t. the strategies O1 and O2.

Strategy	Implemented controls	$i^{c}$	e.i
O1	$\nu_{\phi =1,2}\neq 0$	1.260559486045	0.019394314
O2	$\nu_{1}=0$, $\nu_{2}\neq 0$	1.260559486367	0.019394288

## Numerical simulation and results

In this section, the numerical scheme, simulations, and detailed explanation of the result thus obtained are carried out. Let,


$${ℳ}_{1}^{*}(S,E,V,I,\nu_{\phi =1,2})=(1-\eta )\mu -\alpha_{1}(1-k)IS-b_{1}\nu_{1}(t)S+b_{2}\nu_{2}(t)V,$$

$${ℳ}_{2}^{*}(S,E,V,I,\nu_{\phi =1,2})=\alpha_{1}(1-k)IS+\alpha_{2}IV-\gamma_{1}E,$$

$${ℳ}_{3}^{*}(S,E,V,I,\nu_{\phi =1,2})=-\alpha_{2}IV-b_{2}\nu_{2}(t)V,$$

$${ℳ}_{4}^{*}(S,E,V,I,\nu_{\phi =1,2})=\gamma_{1}E-\gamma_{2}I.$$

Inducing AB-integral to model (10) gives

$$S(t)=S(0)+\frac{\rho t^{\rho -1}{ℳ}_{1}^{*}\left(S,E,V,I,\nu_{\phi =1,2},t\right)}{P(\tau )}\;\;+\rho Q(\tau )\int_{0}^{t}(t-l{)}^{\tau -1}l^{\rho -1}{ℳ}_{1}^{*}\left(S,E,V,I,\nu_{\phi =1,2},l\right)dl,\;E(t)=E(0)+\frac{\rho t^{\rho -1}{ℳ}_{2}^{*}\left(S,E,V,I,\nu_{\phi =1,2},t\right)}{P(\tau )}\;\;+\rho Q(\tau )\int_{0}^{t}(t-l{)}^{\tau -1}l^{\rho -1}{ℳ}_{2}^{*}\left(S,E,V,I,\nu_{\phi =1,2},l\right)dl,\;V(t)=V(0)+\frac{\rho t^{\rho -1}{ℳ}_{3}^{*}\left(S,E,V,I,\nu_{\phi =1,2},t\right)}{P(\tau )}\;\;+\rho Q(\tau )\int_{0}^{t}(t-l{)}^{\tau -1}l^{\rho -1}{ℳ}_{3}^{*}\left(S,E,V,I,\nu_{\phi =1,2},l\right)dl,\;I(t)=I(0)+\frac{\rho t^{\rho -1}{ℳ}_{4}^{*}\left(S,E,V,I,\nu_{\phi =1,2},t\right)}{P(\tau )}\;\;+\rho Q(\tau )\int_{0}^{t}(t-l{)}^{\tau -1}l^{\rho -1}{ℳ}_{4}^{*}\left(S,E,V,I,\nu_{\phi =1,2},l\right)dl.$$ (19)

Further, *t* is replaced by *t*_*n* + 1_ in the integrands of Eq 19 and necessary adjustments elsewhere in the same set of equations to get

$$S^{n+1}(t)=S(0)+\frac{\rho t_{n}^{\rho -1}{ℳ}_{1}^{*}\left(S^{n},E^{n},V^{n},I^{n},\nu_{\phi =1,2}^{n},t_{n}\right)}{P(\tau )}\;\;+\rho Q(\tau )\int_{0}^{t_{n+1}}(t_{n+1}-l{)}^{\tau -1}l^{\rho -1}{ℳ}_{1}^{*}\left(S,E,V,I,\nu_{\phi =1,2},l\right)dl,\;E^{n+1}(t)=E(0)+\frac{\rho t_{n}^{\rho -1}{ℳ}_{2}^{*}\left(S^{n},E^{n},V^{n},I^{n},\nu_{\phi =1,2}^{n},t_{n}\right)}{P(\tau )}\;\;+\rho Q(\tau )\int_{0}^{t_{n+1}}(t_{n+1}-l{)}^{\tau -1}l^{\rho -1}{ℳ}_{2}^{*}\left(S,E,V,I,\nu_{\phi =1,2},l\right)dl,\;V^{n+1}(t)=V(0)+\frac{\rho t_{n}^{\rho -1}{ℳ}_{3}^{*}\left(S^{n},E^{n},V^{n},I^{n},\nu_{\phi =1,2}^{n},t_{n}\right)}{P(\tau )}\;\;+\rho Q(\tau )\int_{0}^{t_{n+1}}(t_{n+1}-l{)}^{\tau -1}l^{\rho -1}{ℳ}_{3}^{*}\left(S,E,V,I,\nu_{\phi =1,2},l\right)dl,\;I^{n+1}(t)=I(0)+\frac{\rho t_{n}^{\rho -1}{ℳ}_{4}^{*}\left(S^{n},E^{n},V^{n},I^{n},\nu_{\phi =1,2}^{n},t_{n}\right)}{P(\tau )}\;\;+\rho Q(\tau )\int_{0}^{t_{n+1}}(t_{n+1}-l{)}^{\tau -1}l^{\rho -1}{ℳ}_{4}^{*}\left(S,E,V,I,\nu_{\phi =1,2},l\right)dl.$$ (20)

Applying Adams–Bashforth method in Eq 20 gives

$$S^{n+1}(t)=S(0)+\frac{\rho t_{n}^{\rho -1}{ℳ}_{1}^{*}\left(S^{n},E^{n},V^{n},I^{n},\nu_{\phi =1,2}^{n},t_{n}\right)}{P(\tau )}\;\;+\rho Q(\tau )\sum_{r=0}^{n}\int_{t_{r}}^{t_{r+1}}(t_{n+1}-l{)}^{\tau -1}l^{\rho -1}{ℳ}_{1}^{*}\left(S,E,V,I,\nu_{\phi =1,2},l\right)dl,\;E^{n+1}(t)=E(0)+\frac{\rho t_{n}^{\rho -1}{ℳ}_{2}^{*}\left(S^{n},E^{n},V^{n},I^{n},\nu_{\phi =1,2}^{n},t_{n}\right)}{P(\tau )}\;\;+\rho Q(\tau )\sum_{r=0}^{n}\int_{t_{r}}^{t_{r+1}}(t_{n+1}-l{)}^{\tau -1}l^{\rho -1}{ℳ}_{2}^{*}\left(S,E,V,I,\nu_{\phi =1,2},l\right)dl,\;V^{n+1}(t)=V(0)+\frac{\rho t_{n}^{\rho -1}{ℳ}_{3}^{*}\left(S^{n},E^{n},V^{n},I^{n},\nu_{\phi =1,2}^{n},t_{n}\right)}{P(\tau )}\;\;+\rho Q(\tau )\sum_{r=0}^{n}\int_{t_{r}}^{t_{r+1}}(t_{n+1}-l{)}^{\tau -1}l^{\rho -1}{ℳ}_{3}^{*}\left(S,E,V,I,\nu_{\phi =1,2},l\right)dl,\;I^{n+1}(t)=I(0)+\frac{\rho t_{n}^{\rho -1}{ℳ}_{4}^{*}\left(S^{n},E^{n},V^{n},I^{n},\nu_{\phi =1,2}^{n},t_{n}\right)}{P(\tau )}\;\;+\rho Q(\tau )\sum_{r=0}^{n}\int_{t_{r}}^{t_{r+1}}(t_{n+1}-l{)}^{\tau -1}l^{\rho -1}{ℳ}_{4}^{*}\left(S,E,V,I,\nu_{\phi =1,2},l\right)dl,$$ (21)

with *t*_0_ = 0. If $\psi =t_{r+1}-t_{r}$, then to discritize the Eq 21, the Adams–Moulton method is used to get (for the iterative scheme we take *t*_*n* + 1_ = 300 and ψ=0.5)

$$S^{n+1}(t)=S(0)+\frac{\rho t_{n}^{\rho -1}{ℳ}_{1}^{*}\left(S^{n},E^{n},V^{n},I^{n},\nu_{\phi =1,2}^{n},t_{n}\right)}{P(\tau )}+\frac{s\psi^{\tau }}{𝒩(\tau )\Gamma (\tau +2)}\;\;\times \sum_{r=1}^{n}[t_{r}^{\rho -1}{ℳ}_{1}^{*}(t_{r},S^{r},E^{r},V^{r},I^{r},\nu_{\phi =1,2}^{r})\times ({\left(n+1-r\right)}^{\tau }\left(n-r+2+\tau \right)\;\;-{\left(n-r\right)}^{\tau }\left(n-r+2+2\tau \right))-t_{r-1}^{\rho -1}{ℳ}_{1}^{*}(t_{r},S^{r-1},E^{r-1},V^{r-1},I^{r-1},\nu_{\phi =1,2}^{r-1})\;\;\times ((n-r+1{)}^{\tau +1}-(n-r{)}^{\tau }(n-r+1+\tau ))],\;E^{n+1}(t)=E(0)+\frac{\rho t_{n}^{\rho -1}{ℳ}_{2}^{*}\left(S^{n},E^{n},V^{n},I^{n},\nu_{\phi =1,2}^{n},t_{n}\right)}{P(\tau )}+\frac{\rho \psi^{\tau }}{𝒩(\tau )\Gamma (\tau +2)}\;\;\times \sum_{r=1}^{n}[t_{r}^{\rho -1}{ℳ}_{2}^{*}(t_{r},S^{r},E^{r},V^{r},I^{r},\nu_{\phi =1,2}^{r})\times ({\left(n+1-r\right)}^{\tau }\left(n-r+2+\tau \right)\;\;-{\left(n-r\right)}^{\tau }\left(n-r+2+2\tau \right))-t_{r-1}^{\rho -1}{ℳ}_{2}^{*}(t_{r},S^{r-1},E^{r-1},V^{r-1},I^{r-1},\nu_{\phi =1,2}^{r-1})\;\;\times ((n-r+1{)}^{\tau +1}-(n-r{)}^{\tau }(n-r+1+\tau ))],\;V^{n+1}(t)=V(0)+\frac{\rho t_{n}^{\rho -1}{ℳ}_{3}^{*}\left(S^{n},E^{n},V^{n},I^{n},\nu_{\phi =1,2}^{n},t_{n}\right)}{P(\tau )}+\frac{\rho \psi^{\tau }}{𝒩(\tau )\Gamma (\tau +2)}\;\;\;\times \sum_{r=1}^{n}[t_{r}^{\rho -1}{ℳ}_{3}^{*}(t_{r},S^{r},E^{r},V^{r},I^{r},\nu_{\phi =1,2}^{r})\times ({\left(n+1-r\right)}^{\tau }\left(n-r+2+\tau \right)\;\;-{\left(n-r\right)}^{\tau }\left(n-r+2+2\tau \right))-t_{r-1}^{\rho -1}{ℳ}_{3}^{*}(t_{r},S^{r-1},E^{r-1},V^{r-1},I^{r-1},\nu_{\phi =1,2}^{r-1})\;\;\times ((n-r+1{)}^{\tau +1}-(n-r{)}^{\tau }(n-r+1+\tau ))],\;I^{n+1}(t)=I(0)+\frac{\rho t_{n}^{\rho -1}{ℳ}_{4}^{*}\left(S^{n},E^{n},V^{n},I^{n},\nu_{\phi =1,2}^{n},t_{n}\right)}{P(\tau )}+\frac{\rho \psi^{\tau }}{𝒩(\tau )\Gamma (\tau +2)}\;\;\times \sum_{r=1}^{n}[t_{r}^{\rho -1}{ℳ}_{4}^{*}(t_{r},S^{r},E^{r},V^{r},I^{r},\nu_{\phi =1,2}^{r})\times ({\left(n+1-r\right)}^{\tau }\left(n-r+2+\tau \right)\;\;-{\left(n-r\right)}^{\tau }\left(n-r+2+2\tau \right))-t_{r-1}^{\rho -1}{ℳ}_{4}^{*}(t_{r},S^{r-1},E^{r-1},V^{r-1},I^{r-1},\nu_{\phi =1,2}^{r-1})\;\;\times ((n-r+1{)}^{\tau +1}-(n-r{)}^{\tau }(n-r+1+\tau ))].$$ (22)

**Remark 4.** *If $\nu_{\phi =1,2}=0$, then the Eqs* 19*–*22 *reduces to the numerical solutions of the model (1), where ${ℳ}_{\phi =1,2,3,4}^{*}$ are replaced by ${ℳ}_{\phi =1,2,3,4}$.*

For the proposed model (1), the initial consideration of state variables are S(0)=0.99 (meaning 99% of the total population are susceptives at initial stage), E(0)=0.80, V(0)=0.05 and I(0)=0.5. The parameters value are taken from Table 1. The COVID-19 infection disappears within a week or two, so short memory effect is associated by implementing fractional parameters closer to unity i.e., τ,ρ∈(0.8,1]. If it is considered that the non-drugs/non-vaccines measure τ,ρ∈(0.8,1] lowers the risk of infection by 60%, 70% and 80%, then its probabilistic values can be taken as 0.6, 0.7 and 0.8, respectively.

**Fig 6:**
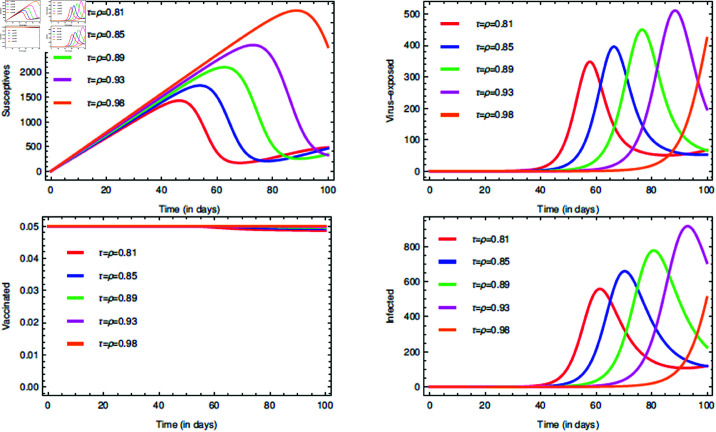
Impact of the fractional parameters τ and ρ on state variables and time series dynamics with no strategies when k=0.8.

**Fig 7:**
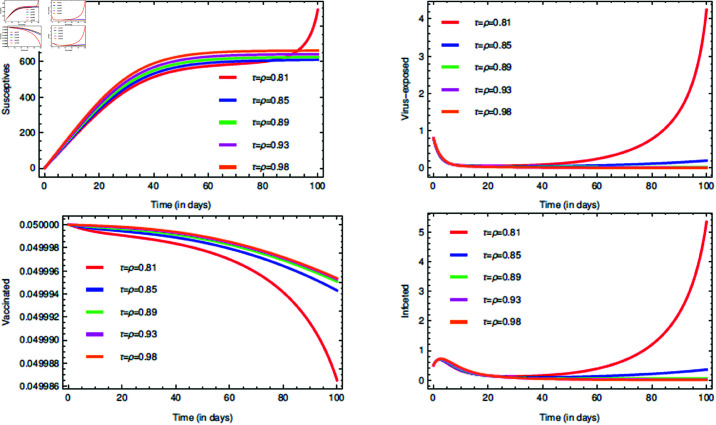
Impact of the fractional parameters τ and ρ on state variables and time series dynamics with strategy O1 when k=0.8.

**Fig 8:**
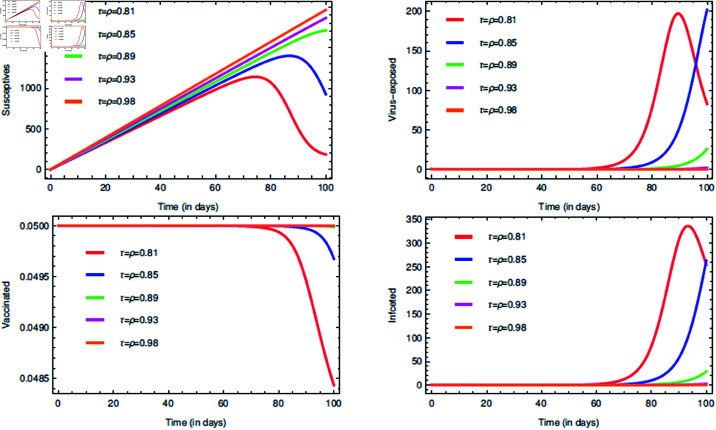
Impact of the fractional parameters τ and ρ on state variables and time series dynamics with strategy O2 when k=0.8.

The Figs 6, 7, and 8 plots the time-series graphs for the state variables varying the fractional parameters τ and ρ under no strategies, strategy O1, and strategy O2, respectively. The dynamics are evaluated when there is 20% chance of risk to acquire infection. The peaks are attained after *t* = 40 for the cases where no strategies are applied and startegy O1, where as for strategy O2 the peaks are attained after *t* = 60. The infection rate due to poor environment, $\alpha_{1}$, is associated with the state variables *S* and *E*, and also times $t_{\phi =1,2}$. In Fig 6, when no strategies are implemented, it is seen that the infection in susceptible class increases till the time *t* = 30 where as the virus-exposed populace and infected populace remains constant. When strategy O1 is implemented, it is seen that the system tends to vaccine-clearance equilibrium point ${𝔹}_{0}$ after time *t* = 80 (see Fig 7). In all this when strategy O2 is taken under consideration, infection gets reduced after *t* = 80 and then the equilibrium point ${ℬ}_{0}$ can be attained (see Fig 8).

**Fig 9:**
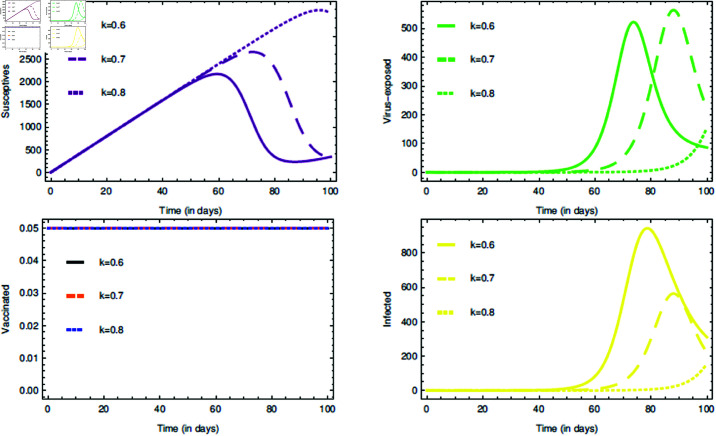
Impact of the rate k on state variables and time series dynamics.

**Fig 10:**
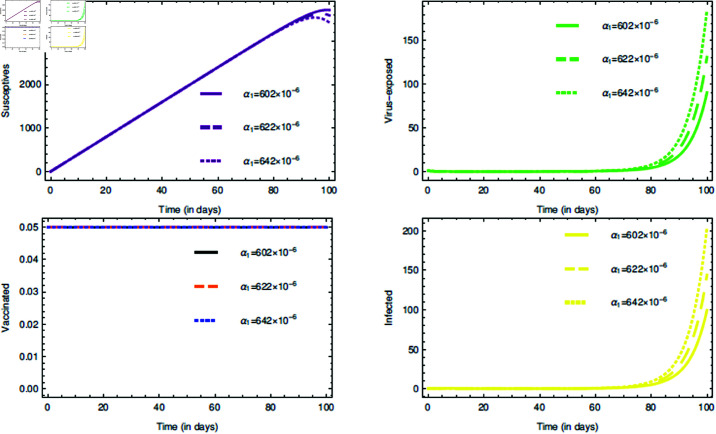
Impact of the rate $\alpha_{1}$ on state variables and time series dynamics.

The Fig 9 plots the time series graphs for the state variables by varying *k*. Its affect can be seen in the trajectories of state variables in mid-way of the time period, except for the vaccinated individuals. This is because once the drug/vaccine is administered in the body, the immune-system battles with the virus keeping the environment health as redundant. According to the proposed model’s hypothetical assumption, infection can also ocurr even in 80% cleaner habitat and therefore *k* = 0.8 is fixed for further interventions. The rate $\alpha_{1}$ is varied and in Fig 10 it can be seen that there are changes in the dynamics of state variables, but very minute. This proves the fact that $\alpha_{1}$ is very highly sensitive. The change can be seen in the end of the time period. This also justifies our assumption about the mechanisms of COVID-19 transmission as discussed in section “The model: Formulation and assumptions". The path tarjectories of virus-exposed and infected individuals are bit same. The justification for Fig 10c is quite simillar to that of Fig 9c.

**Fig 11:**
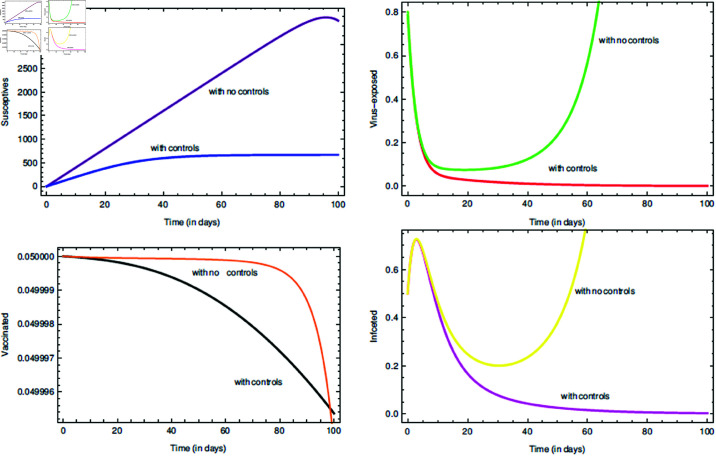
Impact of the controls $\nu_{\phi =1,2}$ on state variables and time series dynamics.

**Fig 12:**
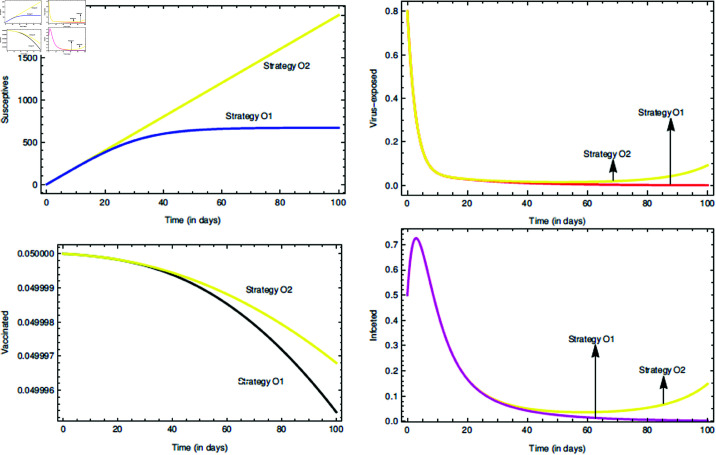
Efficient roles of the strategies O1 and O2 on state variables and time series dynamics.

For the proposed model (10), the initial consideration of state variables remains the same. To conduct the numerical scheme, τ,ρ=0.95 [46] are taken, and the other parameter values can be found in Table 1 together with $\alpha_{2}=10^{-7}$. The Fig 11 depicts about the two incorporated control functions given in model (10). In an environment with 80% cleaner habitat, it is seen that the controls $\nu_{1}$ and $\nu_{2}$ can contain the infection spread and bring change in all the state variable trajectories. For the numerical simulation part the parameters value are hypothetically taken as: *A*_1_ = 5, *A*_2_ = 10, *B*_1_ = 15, *B*_2_ = 20, *b*_1_ = 0.002 [76] and *b*_2_ = 0.001 [76]. As discussed in the subsection “Numerical explanation of the controls and Efficiency index", Fig 12 describes about exercising the strategies, viz. O1 and O2 on the COVID-19 SEVI model (1). It is clear that strategy O1 is better than that of O2, but from the economic point of view strategy O2 is efficient. Thus, it can be said that the graphical results show notable impact of the two controls.

**Fig 13:**
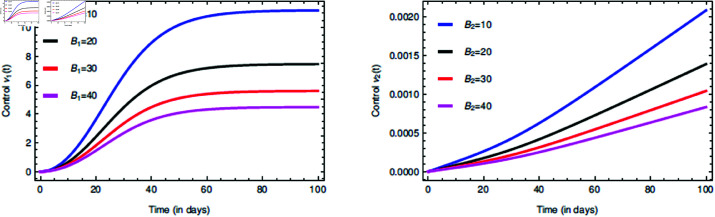
Time series dynamics of the controls $\nu_{\phi =1,2}$ for their various respective weight parameters $B_{\phi =1,2}$.

The Fig 13 explains about the role of the weight parameters $B_{\phi =1,2}$ in the two controls $\nu_{\phi =1,2}$. This suggests that on some critical conditions during the infection spread how the controls can be made much more effective by changing their way of handling.

## Discussions

Apart from the vaccinated individuals, all other state variables are highly influenced by the measure *k* which depends on the environment health. This supports the statement that rate of infection spread depends on the hygiene of an individual’s surroundings. But it is also true that only preventive measures cannot contain the infection spread. Even though the short-term benefits of COVID-19 environmental quality were first seen as a “window of opportunity" [77], further research is necessary to fully understand the long-term implications of the virus for ecological sustainability, climate action, and ecosystem resilience. Therefore, to control its spread dynamics, geographical factors can also affect the factors controlling the dispersion of air pollutants or meteorological conditions. The fractional parameters, τ and ρ are two crucial factors involved in your COVID-19 model; they possess important mathematical effects on the dynamics of the model and its stability; moreover, they have far-reaching biological effects on the features of COVID-19 and on the means of combating the disease. Managing and controlling these parameters is critical to the containment of diseases and the reduction of their impact.

Since the parameter $\alpha_{1}$ is highly sensitive, therefore hygienic environment is a must. Neighbourhood ecosystem cleaning can be done using disinfectants like sodium hypochlorite, peracetic acid, etc., as well as ultraviolet light. On the other hand, using these oxidants widely to manage COVID-19 may have unfavorable side effects. For instance, using sodium hypochlorite excessively can result in high concentrations of disinfectant byproducts, which can then be converted into even more dangerous halogenated organic compounds [78]. In order to help mitigate health risks caused by COVID-19 infections, appropriate epidemiological studies must be conducted in order to identify vulnerable groups based on air quality factors, climate and environmental data, and other relevant factors [79]. Because the effects of the disease on climate are unpredictable, it is important to investigate the potential long-term effects of such aspects. Hence, for incorporated COVID-19 management, mitigation, livelihood assurance, and human well-being, it is imperative to pragmatically identify trade-offs among sustainability concerns and the nexus approach. The incorporated COVID-19 related steps can only be determined that must be taken to reduce risks to eco-sociological and socio-economical systems with a holistic nexus strategy. To lessen the effects of the epidemic, the interconnectedness of human health, climate change, energy, water and food security, social justice, and livelihood should also be addressed in an integrated manner.

Analyzing the stability of the vaccine-clearance equilibrium point turns into the focus of attention. Epidemiology helps us forecast whether the new round of the vaccination shall culminate in the total elimination of the disease or it shall reach a stable incidence. Stability analysis can be useful for forming the public health post vaccination trajectories insights for the separate factors that may affect the long term results. Such features include the effectiveness of the vaccines used, the proportion of the population that has been vaccinated, as well as the history of the infection. Not only abiding to govt. policies but awareness campaining is also necessary.

Lock-downs had a detrimental impact on livelihoods by upsetting agricultural economies and activities. It has been reported that during COVID-19, indigenous rural populations experienced severe socio-economic distress as a result of losing their livelihoods and having restricted availability of their food resources. Modified food crops, however, can be expensive and infrequently reach rural populations due to their fortified immunity-boosting nutrients [80]. It is necessary to provide context for the detrimental effects of COVID-19 on livelihoods for which the agriculture and traditional food sectors must be strengthened to guarantee human health. Researchers can analyze a wide range of scenarios and evaluate the potential effects of various strategies, like adding more hospitals or implementing social distancing measures, which helps with the prevention and mitigation of epidemics. Real-time data-driven computational frameworks can update and change estimates, which may have an impact on policy choices and public health initiatives. Several mathematical epidemiology researchers have written articles about F-F operators. A new method for examining the dynamics of disease and its spread across populations is provided by the proposed F-F model. Under the influence of fractal geometry and fractional calculus, the models (1) and (10) sheds light on the intricate and interrelated structure of disease spread. It is seen that COVID-19 awareness campaign and vaccination drive can bring some significant changes. Though strategy O2 is economically beneficial but new methods need to be implemented for strategy O1 to function economically as awareness is must.

## Conclusions

In this study, a novel F-F order COVID-19 SEVI models Figs 1 and 10 are proposed and compared to investigate both the dynamics of COVID-19 and the potential interventions on the disease progression. Due to the method of fractional calculus, it was possible to take into account the numerous and multistage processes occurring in the systems, which classical models do not reveal and therefore significantly improve the accuracy of the calculations. Given such an approach, it was possible to accurately assess the potential of the virus infection process and make judgments about its impact on people and the external environment.

Our study demonstrates the impact of the epidemic response in terms of the environment pointing at shifts in ecological factors attributed to people’s behavior and implemented countermeasures. Epidemiology can thus be said to be intertwined with environmental science in that there is the need to employ multiple approaches when it comes to the management of public health issues. Recognizing these connections is crucial because it opens the possibility to develop better, and more durable strategies for fighting epidemics. The control measures invoking the proposed model like awareness campaign, and vaccination drive amongst other non-pharmacological interventions has been discussed. It let us create better policies, taking into view long-term consequences of some measures to for the population. These discoveries may equip the policy makers with information that will assist them in addressing the needs of the public especially on health while protecting the environment.

The true innovation of our work lies in the application of fractional calculus to the SEVI model. This mathematical approach provided a deeper and more nuanced understanding of COVID-19 dynamics by incorporating Adams–Bashforth–Moulton method. It offers a new way to look at epidemic processes, enhancing the precision and effectiveness of control measures. The research article demonstrates the powerful potential of fractional calculus in improving our understanding of complex epidemiological models. By highlighting the environmental impacts and developing more effective control strategies, the F-F order SEVI models provides a vital tool for future studies. This work encourages a more integrated approach to public health and environmental policy, paving the way for innovative solutions in managing infectious diseases and their broader ecological consequences.

More tangential research could be done to describe various forms of economic impact regarding different control measures such as the cost and efficacy of organising lockdowns, vaccinations, and others. This could be of importance especially to policymakers in making the appropriate decisions and vivian also recommends that course based. One can suppose that expanding on the existing model and taking into account the environmental and societal components can show how various communities suffer the epidemic or, on the contrary, resist it. This could help in targeting some of the population needs for intervention in a way that respects demographic and geographical characteristics. Focusing more on the state of art of the optimal control using machine learning and Artificial Intelligence to improve the interventions. That of course would require creation of program that would continuously adjust how control measures are being implemented depending on conditions present in an epidemic. A more general comparative mathematical analysis can be performed using our proposed techniques and the methods dicsussed in the references [65, 81].

### Ethics statement.

During the research, this article does not contain any kind of experiments with humans as well as animals by the authors.
